# Serological Evidence of Widespread Circulation of West Nile Virus and Other Flaviviruses in Equines of the Pantanal, Brazil

**DOI:** 10.1371/journal.pntd.0002706

**Published:** 2014-02-13

**Authors:** Alex Pauvolid-Corrêa, Zilca Campos, Raquel Juliano, Jason Velez, Rita Maria Ribeiro Nogueira, Nicholas Komar

**Affiliations:** 1 Laboratório de Flavivírus, Instituto Oswaldo Cruz, Fundação Oswaldo Cruz, Ministério da Saúde, Pavilhão Helio e Peggy Pereira, Rio de Janeiro, Rio de Janeiro, Brazil; 2 Arbovirus Diseases Branch, Centers for Disease Control and Prevention, Fort Collins, Colorado, United States of America; 3 Fulbright Visiting Researcher in Doctorate Sandwich Program at Centers for Disease Control and Prevention, Fort Collins, Colorado, United States of America; 4 Embrapa Pantanal, Ministério da Agricultura Pecuária e Abastecimento, Corumbá, Mato Grosso do Sul, Brazil; Florida Gulf Coast University, United States of America

## Abstract

A recent study reported neutralizing antibodies to West Nile virus (WNV) in horses from four ranches of southern Pantanal. To extend that study, a serosurvey for WNV and 11 Brazilian flaviviruses was conducted with 760 equines, 238 sheep and 61 caimans from 17 local cattle ranches. Among the tested equines, 32 were collected from a ranch where a neurologic disorder outbreak had been recently reported. The sera were initially screened by using a blocking ELISA and then titrated by 90% plaque-reduction neutralization test (PRNT_90_) for 12 flaviviruses. Employing the criterion of 4-fold greater titer, 78 (10.3%) equines were seropositive for Ilheus virus, 59 (7.8%) for Saint Louis encephalitis virus, 24 (3.2%) for WNV, two (0.3%) for Cacipacore virus and one (0.1%) for Rocio virus. No serological evidence was found linking the neurological disease that affected local equines to WNV. All caimans and sheep were negative by blocking ELISA for flaviviruses. There were no seropositive equines for Bussuquara, Iguape, Yellow fever and all four Dengue virus serotypes. The detection of WNV-seropositive equines in ten ranches and ILHV and SLEV-seropositive equines in fourteen ranches of two different sub-regions of Pantanal is strong evidence of widespread circulation of these flaviviruses in the region.

## Author Summary

West Nile virus is maintained in cycles between birds and mosquitoes and recently reemerged as a worldwide major public health and veterinarian concern as the cause of human and equine encephalitis outbreaks. Recent studies have reported serological evidence of West Nile virus circulation in Pantanal, west-central region of Brazil. However, considering the co-circulation of various cross-reactive flaviviruses in Brazil and that most of the flaviviruses isolated in Brazil are unknown or understudied in the Pantanal, serological results should be interpreted with caution. Therefore, we conducted a serosurvey for West Nile virus and the 11 Brazilian flaviviruses of potential medical importance, utilizing equines, sheep and caimans as indicators, including 32 equines collected from a ranch where a neurologic disorder had been recently reported among the equines. We found serological evidence of Ilheus, Saint Louis encephalitis, West Nile, Cacipacore and Rocio viruses in Pantanal equines. West Nile virus infection was not associated with the neurological disease of equines. The detection of WNV-seropositive equines in ten ranches and ILHV- and SLEV-seropositive equines in fourteen ranches of two different sub-regions of Pantanal is strong evidence of widespread circulation of these flaviviruses in the region.

## Introduction

Flaviviruses represent a group of mosquito-borne viruses in Brazil that are annually involved in a large number of human cases of dengue fever countrywide and sporadic local outbreaks of sylvatic yellow fever [1], [2]. Outbreaks caused by other flaviviruses have also been reported in the country. In the 1970s, the largest Brazilian epidemic of arbovirus encephalitis was caused by Rocio virus (ROCV) in southeast Brazil [3]. More recently, an outbreak of hemorrhagic manifestations was linked to Saint Louis encephalitis virus (SLEV) [4]. Sporadic human cases caused by other sylvatic flaviviruses, including Bussuquara virus (BSQV), Ilheus virus (ILHV) and Cacipacore virus (CPCV) have also been reported in Brazil [5], [6], [7]. Furthermore, yellow fever epizootics in howler monkeys were reported in 2008 and 2009. Approximately 200 carcasses tested positive for Yellow fever virus (YFV) and about 2000 deaths were reported [8].

Thirteen flaviviruses have been reported in Brazil, listed here in chronological order of discovery: YFV, ILHV, BSQV, SLEV, ROCV, CPCV, Dengue virus 1 (DENV-1) and Dengue virus 4 (DENV-4), Dengue virus 2 (DENV-2), Iguape virus (IGUV), Naranjal-like virus (NJLV), Dengue virus 3 (DENV-3) and Culex flavivirus (CXFV) [9], [10], [11], [12], [13], [14], [15], [16], [17], [18], [19], [20]. In 2009, serological evidence of West Nile virus (WNV) infection in Brazilian horses, was collected for the first time, in the Pantanal wetland region of Mato Grosso do Sul state (MS) [21].

The Pantanal wetland is a subtropical region of great biodiversity with strong potential for maintenance and evolution of mosquito-borne viruses. Comprising approximately 140,000 km^2^, the Pantanal is a vast sedimentary floodplain characterized by seasonal flooding which determines specific ecosystem processes, with the occurrence of plants and animals that are adapted to the annual shrinking and expansion of habitats due to the seasonal hydrological regime [22]. The region, which covers mainly Brazilian but also Paraguayan and Bolivian territories, is ecologically classified into 11 sub-regions according to vegetation, flooding and physiography. In Brazil, the Pantanal is located within the states of Mato Grosso (MT) and MS in the west-central region of the country [23].

Most of the flaviviruses isolated in Brazil are unknown or understudied in the Pantanal. A small number of investigations in the Nhecolândia Sub-region of the Pantanal, MS, have detected serological evidence for four flaviviruses, including ILHV, SLEV, WNV and CPCV [21], [24], [25]. Recently, three other serological studies reported the detection of neutralizing antibodies for WNV in equines from MT and again in MS, including from outside the boundaries of the Pantanal [26], [27], [28]. Complete surveys for flaviviruses in the Pantanal have not been reported. Therefore, we conducted a serosurvey for WNV and the 11 Brazilian flaviviruses of potential medical importance, utilizing equines, sheep and caimans as indicators.

## Materials and Methods

**Figure 1:**
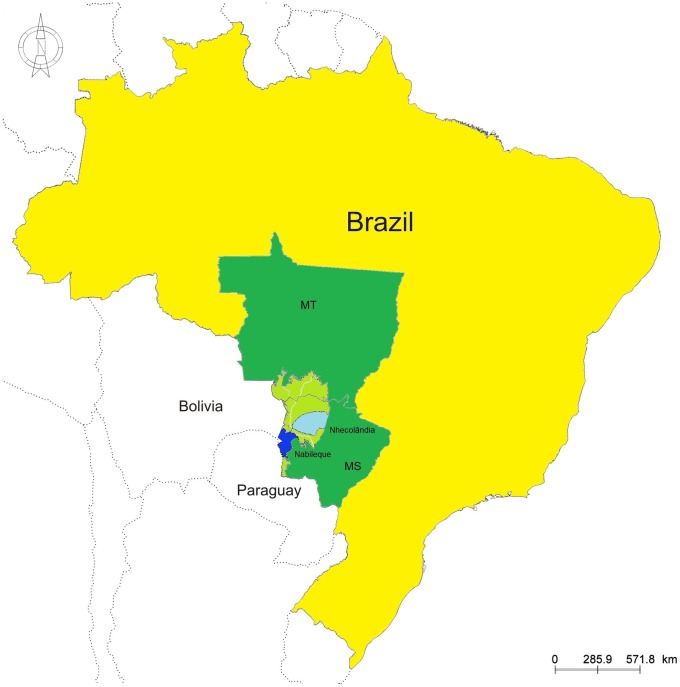
Pantanal located in west-central Brazil. Pantanal located within the states of Mato Grosso (MT) and Mato Grosso do Sul (MS) is shown in light green. Nhecolândia and Nabileque sub-regions, where a serosurvey for flaviviruses was conducted in equines, sheep and caimans in 2009 and 2010 are shown in light and dark blue respectively.

In October 2009, April, September and October 2010, blood samples were taken from 724 equines, 238 sheep and 61 caimans from 16 beef cattle ranches comprising an area of approximately 2,700 square-km in the Nhecolândia Sub-region of Pantanal, MS, west-central Brazil (Figure 1).

### Ethics statement

The collections for this study were approved by the Animal Ethics Committee of Fundação Oswaldo Cruz (License CEUA-Fiocruz LW-1/12, protocol P-74/10-5) in compliance with the requirements of Brazilian Law 11794/2008, which rules on the scientific use of animals, including the principles of the Brazilian society of Science in laboratory animals. The collections were also approved by the Brazilian Institute of Environment and Natural Resources (licenses IBAMA 18363-1/2009 and 18363-2/2010).

Caimans were captured in two ranches from sites where a high concentration of these animals was observed. Caiman blood samples were obtained by puncture of the internal jugular vein between the 1st and 2nd cervical vertebrae as described previously [29]. Information recorded about caimans included gender, weight and snout-vent length.

Blood samples from equines, including horses, donkeys and mules, and from sheep were taken by puncture of the jugular vein in 14 and nine different ranches, respectively. Gender, age, tameness, breed, vaccination status, travel history outside of Pantanal and history of abnormalities, such as clinical signs involving the central or peripheral nervous system, were recorded for each equine sample. Information about gender, age, breed and travel history outside of Pantanal were also recorded for each sheep. All animals sampled appeared healthy except for one horse that presented with clinical signs suggestive of neurological disorder.

Additionally, 36 horse samples collected in February 2010 from a ranch comprising roughly a 900-square-km area in the Nabileque Sub-region of Pantanal, also located in MS (Figure 1) were tested. An outbreak of an undiagnosed neurological syndrome had caused the death of 16 equines at this location from December 2009 to January 2010. All equines here were vaccinated for rabies and tested negative for *Trypanosoma evansi*, a common pathogen involved in incoordination and instability of hind limbs in Pantanal equines. These equines had not been vaccinated for equine encephalitis viruses at the time of the outbreak. After the first deaths, the herd was vaccinated for eastern and western equine encephalitis viruses.

All samples were initially screened for flavivirus-reactive antibodies by blocking ELISA as described previously [30]. Briefly, the ability of the sera to block the binding of the flavivirus group-reactive monoclonal antibody 6B6C-1 to the cell lysate-derived antigen for WNV was compared to the blocking ability of negative control horse serum. Samples were considered seropositive when the inhibition values produced were ≥30%. Samples with inhibition values between 19 and 29% were repeated twice to confirm the negative results. Blocking ELISA-positive samples were then heat-inactivated and tested by PRNT_90_, as previously described [31], for WNV and 11 Brazilian flaviviruses. Briefly, serial two-fold dilutions that ranged from 1∶10–1∶320 or 1∶20–1∶640 of each blocking ELISA seropositive sample were tested for their ability to neutralize plaque formation by WNV, SLEV, ILHV, CPCV, BSQV, IGUV, YFV (vaccine strain 17D), ROCV, DENV-1, DENV-2, DENV-3 (recombinant ChimeriVax Dengue 3 virus) and DENV-4. Caiman and sheep samples that were negative by blocking ELISA were also tested by PRNT_90_ for WNV.

All reference viruses used for PRNT were previously submitted to partial nucleotide sequencing of the N terminal region of NS5 gene to confirm viral identity. High identity scores were obtained with the following sequences deposited at GenBank: DENV-1 (FJ562106), DENV-2 (GQ398257), DENV-3 (recombinant ChimeriVax Dengue 3 virus) (JN811143), DENV-4 (GQ199880), ROCV (AY632542), SLEV (EF158048), ILHV (AY632539), YFV (JN628279), BSQV (AY632536), CPCV (AF013367), IGUV (AY632538), and WNV (JN819325).

Serum was considered seropositive to a virus when it reduced at least 90% of the formation of plaques of this virus and its reciprocal neutralizing antibody titer was at least four-fold greater than what was observed for the other 11 tested flaviviruses. The PRNT is the most specific serological test for the differentiation of flavivirus infections in convalescent serum samples [32]. Type-specific antibodies against flaviviruses can be distinguished using the PRNT [33]. The conservative criterion of four-fold greater titer is based on the results of cross-neutralization tests with flaviviruses and their respective antisera used for studies of antigenic relationships [34], [35]. Two or more flaviviruses are distinct from each other by quantitative serological criteria (four-fold or greater differences between homologous and heterologous titers of both serum samples) [32]. The Cochran-Armitage Trend Test was used to test for positive trends in seroprevalence among age classes (StatXact 10.0, Cytel Software Corporation, Cambridge, MA)

## Results

All caiman and sheep serum samples were negative for flaviviruses-specific antibodies using the 6B6C-1 monoclonal antibody by blocking ELISA. When tested by PRNT_90_ for WNV, all caiman samples confirmed negative. Sixty caiman samples had PRNT_90_ titer <10 and one <40 for WNV (low sample volume of the latter required testing at a lowest dilution of 1∶40). From 238 sheep samples tested, 235 had PRNT_90_ titers <10, two showed low neutralizing antibodies titers of 20 and 10 and one <40. From 760 equine samples initially screened for flavivirus-reactive antibodies by blocking ELISA, 396 (52.1%) were positive. When this subset was tested using PRNT_90_, 332 (43.7%) had neutralizing reactivity (PRNT_90_ titer ≥10) for ILHV, 251 (33%) for SLEV, 172 (22.6%) for WNV, 139 (18.3%) for CPCV, 130 (17.1%) for ROCV, 62 (8.2%) for IGUV, 14 (1.8%) for YFV, 12 (1.6%) for BSQV, four (0.5%) for DENV-1, three (0.4%) for DENV-2, one (0.1%) for DENV-4 and none for DENV-3 (Table 1). Employing the criterion of 4-fold greater PRNT_90_ titer, 77 (10.1%) equines were seropositive for ILHV, 59 (7.8%) for SLEV, 24 (3.2%) for WNV (see Table 2), two (0.3%) for CPCV and one (0.1%) for ROCV. From the remaining 232 blocking ELISA positive samples, 227 (29.9%) were considered seropositive for undifferentiated flavivirus due to cross-reactivity and five (0.6%) were considered seronegative with neutralizing antibodies titers <20 or <10 for all of the flaviviruses that were assayed by PRNT_90_. These five may represent infections by an unknown flavivirus.

**Table 1:** Results of PRNT_90_ for WNV and Brazilian flaviviruses in equines (n = 760) of Pantanal, Brazil.

Flaviviruses tested	Number of equines PRNT_90_ titer ≥10 (%)		Number of equines seropositive by PRNT_90_ using four-fold greater titer criterion (%)	
ILHV	332	(43.7)	78	(10.1)
SLEV	251	(33)	59	(7.8)
WNV	172	(22.6)	24	(3.2)
CPCV	139	(18.3)	2	(0.3)
ROCV	130	(17.1)	1	(0.1)
IGUV	62	(8.2)	0	(0)
YFV	14	(1.8)	0	(0)
BSQV	12	(1.6)	0	(0)
DENV-1	4	(0.5)	0	(0)
DENV-2	3	(0.4)	0	(0)
DENV-3	0	(0)	0	(0)
DENV-4	1	(0.1)	0	(0)

BSQV: Bussuquara virus; CPCV: Cacipacore virus; ILHV: Ilheus virus; ROCV: Rocio virus; SLEV: Saint Louis encephalitis virus; WNV: West Nile virus; IGUV: Iguape virus; YFV: Yellow fever virus; DENV: Dengue virus.

**Table 2:** Comparative PRNT_90_ titers for 12 flaviviruses among 24 WNV-seropositive equines of the Pantanal, Brazil.

ID	Age	Ranch	Sub-region	ELISA	WNV	SLEV	ROCV	ILHV	YFV	IGUV	BSQV	CPCV	DENV-1	DENV-2	DENV-3	DENV-4
316	UNK	PH	Nhecolândia	35.5%	160	<20	<10	<10	<10	<10	<10	<10	<10	<10	<10	<10
327	UNK	PH	Nhecolândia	65.8%	40	<20	<10	10	<10	<10	<10	<10	<10	<10	<10	<10
394	UNK	PP	Nhecolândia	56.4%	160	40	<10	<10	<10	<10	<10	<10	<10	<10	<10	<10
422	UNK	PP	Nhecolândia	37.6%	40	<20	<10	<10	<10	<10	<10	<10	<10	<10	<10	<10
461	6 years	PN	Nhecolândia	73.3%	80	<20	<10	20	<10	<10	<10	<10	<10	<10	<10	<10
664	7 years	PR	Nhecolândia	53.0%	40	<20	<10	<10	<10	<10	<10	<10	<10	<10	<10	<10
687	5 years	PI	Nhecolândia	48.7%	80	<20	<10	20	<10	<10	<10	<10	<10	<10	<10	<10
755	8 years	PJ	Nhecolândia	54.5%	160	20	<10	10	<10	<10	<10	<10	<10	<10	<10	<10
786	4 years	PJ	Nhecolândia	48.8%	40	<20	<10	<10	<10	<10	<10	<10	<10	<10	<10	<10
795	2 years	PJ	Nhecolândia	63.8%	80	20	10	10	<10	<10	<10	<10	<10	<10	<10	<10
807	UNK	PJ	Nhecolândia	54.7%	160	<20	<10	10	<10	<10	<10	10	<10	<10	<10	<10
827	8 years	PG	Nhecolândia	45.7%	160	<20	<10	<10	<10	<10	<10	10	<10	<10	<10	<10
828	UNK	PG	Nhecolândia	38.7%	160	40	<10	20	<10	<10	<10	40	<10	<10	<10	<10
830	UNK	PG	Nhecolândia	78.1%	160	40	<10	40	<10	10	<10	10	<10	<10	<10	<10
846	UNK	PG	Nhecolândia	45.4%	40	<20	<10	<10	<10	<10	<10	<10	<10	<10	<10	<10
879	10 years	PG	Nhecolândia	74.8%	80	20	<10	20	<10	<10	<10	<10	<10	<10	<10	<10
883	10 years	PG	Nhecolândia	62.1%	160	40	<10	10	<10	<10	<10	10	<10	<10	<10	<10
890	7 years	PG	Nhecolândia	59.0%	160	20	<10	<10	<10	<10	<10	10	<10	<10	<10	<10
906	UNK	PD	Nhecolândia	47.4%	320	40	<10	10	<10	<10	<10	20	<10	<10	<10	<10
917	UNK	PD	Nhecolândia	39.5%	40	<20	<10	<10	<10	<10	<10	<10	<10	<10	<10	<10
921	UNK	PD	Nhecolândia	68.1%	160	20	<10	20	<10	10	<10	40	<10	<10	<10	<10
940	3 years	PC	Nhecolândia	53.2%	40	<20	<10	<10	<10	<10	<10	10	<10	<10	<10	<10
1080	UNK	PV	Nabileque	58.1%	320	80	<10	20	<10	<10	<10	40	<10	<10	<10	<10
1088	UNK	PV	Nabileque	71.6%	40	<20	<10	10	<10	10	<10	<10	<10	10	<10	<10

BSQV: Bussuquara virus; CPCV: Cacipacore virus; ILHV: Ilheus virus; ROCV: Rocio virus; SLEV: Saint Louis encephalitis virus; WNV: West Nile virus; IGUV: Iguape virus; YFV: Yellow fever virus; DENV: Dengue virus; UNK: Unknown.

There were no seropositive equines using the four-fold antibody titer difference criterion for BSQV, YFV, IGUV, DENV-1, DENV-2, DENV-3 and DENV-4. Monotypic neutralization reactions (i.e. a sample that reacted with only one of the 12 flaviviruses in the panel) were detected for ILHV (n = 27), SLEV (n = 11) and WNV (n = 6).

The only horse from Nhecolândia Sub-region that presented neurological signs was seropositive for undifferentiated flavivirus with the following PRNT_90_ titers: 80 for SLEV, 40 for ILHV, 20 for YFV, 10 for ROCV and <10 for all other flaviviruses tested.

**Figure 2:**
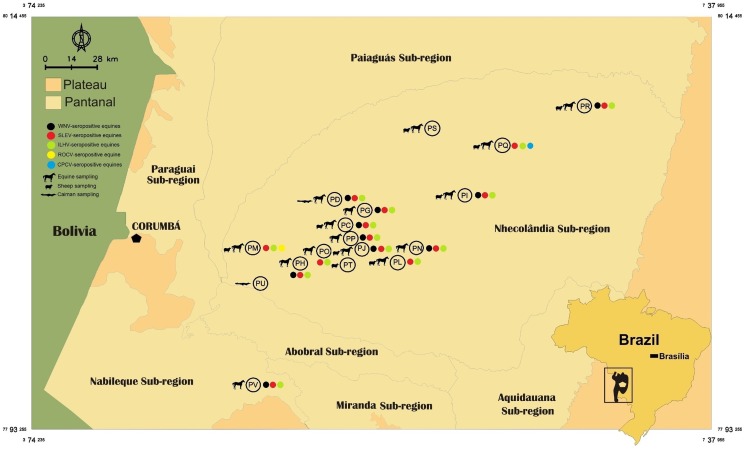
Pantanal ranches where equines, sheep and caimans were surveyed for flaviviruses in the Pantanal. Colored dots mark ranches where seropositive equines with four-fold greater PRNT_90_ titer were detected.

Neutralizing antibodies for WNV were detected in 172 equines from all 15 ranches that equines were tested. Considering the four-fold higher titer criterion as confirmation, the 24 confirmed WNV-seropositive equines were detected in ten ranches comprising an area of approximately 2,500 square-km in the Nhecolândia and Nabileque sub-regions of the Pantanal, located in MS (Figure 2). Besides the 24 WNV-seropositive equines, samples from 37 other equines presented PRNT_90_ titers for WNV ≥40 and among them 26 showed equal or higher titers for one of the other flaviviruses tested. For these 37 equines, the serology profile could not be interpreted to determine whether WNV reactivity was due to infection with WNV or cross-reactivity from other flavivirus infections. In fact, six of them were considered seropositive for one of the 11 other flavivirus tested (Table 3). Low level of neutralizing antibodies was detected for YFV, BSQV, IGUV, DENV-1, DENV-2 and DENV-4 even though none of the equines were considered infected with these viruses. In some cases, the PRNT_90_ titers for YFV, BSQV and IGUV exceeded 20.

**Table 3:** PRNT_90_ titers in WNV-seronegative equines that had titers ≥40 for WNV.

ID	Ranch	Sub-region	WNV	SLEV	ROCV	ILHV	DENV-1	YFV	IGUV	BSQV	CPCV	DENV-1	DENV-2	DENV-3	DENV-4	RESULT
377	PL	Nhecolândia	40	40	<10	10	<10	<10	<10	<10	<10	<10	<10	<10	<10	FLAV
319	PH	Nhecolândia	40	<20	<10	40	<10	<10	<10	<10	10	<10	<10	<10	<10	FLAV
900	PD	Nhecolândia	40	40	<10	<10	<10	<10	<10	<10	20	<10	<10	<10	<10	FLAV
406	PP	Nhecolândia	40	20	<10	40	<10	<10	10	<10	10	<10	<10	<10	<10	FLAV
450	PN	Nhecolândia	40	20	10	10	<10	<10	<10	<10	40	<10	<10	<10	<10	FLAV
672	PI	Nhecolândia	40	<20	<10	40	<10	<10	<10	10	<10	<10	<10	<10	<10	FLAV
681	PI	Nhecolândia	40	80	<10	10	<10	<10	<10	<10	10	<10	<10	<10	<10	FLAV
822	PG	Nhecolândia	40	160	<10	320	<10	<10	<10	<10	10	<10	<10	<10	<10	FLAV
824	PG	Nhecolândia	40	40	10	20	<10	<10	10	<10	10	<10	<10	<10	<10	FLAV
842	PG	Nhecolândia	40	80	<10	10	<10	<10	<10	<10	40	<10	<10	<10	<10	FLAV
691	PJ	Nhecolândia	40	80	40	40	<10	<10	10	<10	80	<10	<10	<10	<10	FLAV
693	PJ	Nhecolândia	40	20	10	40	<10	<10	10	<10	20	<10	<10	<10	<10	FLAV
732	PJ	Nhecolândia	40	<20	<10	40	<10	<10	10	<10	20	<10	<10	<10	<10	FLAV
452	PN	Nhecolândia	40	<20	<10	160	<10	<10	10	<10	10	<10	<10	<10	<10	ILHV
692	PJ	Nhecolândia	40	40	10	320	<10	<10	10	10	20	<10	<10	<10	<10	ILHV
722	PJ	Nhecolândia	40	<20	<10	160	<10	<10	<10	<10	10	<10	<10	<10	<10	ILHV
357	PM	Nhecolândia	40	40	160	40	<10	<10	10	<10	10	<10	<10	<10	<10	ROCV
775	PJ	Nhecolândia	40	640	<10	10	<10	<10	<10	<10	80	<10	<10	<10	<10	SLEV
331	PH	Nhecolândia	80	<20	10	80	<10	10	10	<10	160	<10	<10	<10	<10	FLAV
453	PN	Nhecolândia	80	<20	<10	80	<10	<10	10	<10	<10	<10	<10	<10	<10	FLAV
843	PG	Nhecolândia	80	40	40	80	<10	<10	10	20	80	<10	<10	<10	<10	FLAV
891	PG	Nhecolândia	80	<20	<10	80	<10	<10	10	<10	<10	<10	<10	<10	<10	FLAV
750	PJ	Nhecolândia	80	160	<10	40	<10	<10	<10	<10	80	<10	<10	<10	<10	FLAV
803	PJ	Nhecolândia	80	80	40	40	10	<10	10	<10	<10	10	<10	<10	<10	FLAV
804	PJ	Nhecolândia	80	20	<10	80	<10	<10	<10	<10	10	<10	<10	<10	<10	FLAV
863	PG	Nhecolândia	80	40	<10	320	<10	<10	<10	<10	20	<10	<10	<10	<10	ILHV

BSQV: Bussuquara virus; CPCV: Cacipacore virus; ILHV: Ilheus virus; ROCV: Rocio virus; SLEV: Saint Louis encephalitis virus; WNV: West Nile virus; IGUV: Iguape virus; YFV: Yellow fever virus; DENV: Dengue virus.

Both ILHV- and SLEV-seropositive equines were detected in 14 of the 15 ranches that equines were tested, comprising roughly a 3,100-square-km area. The only ranch that had no ILHV- and SLEV-seropositive equines was ranch PS (Figure 2).

**Figure 3:**
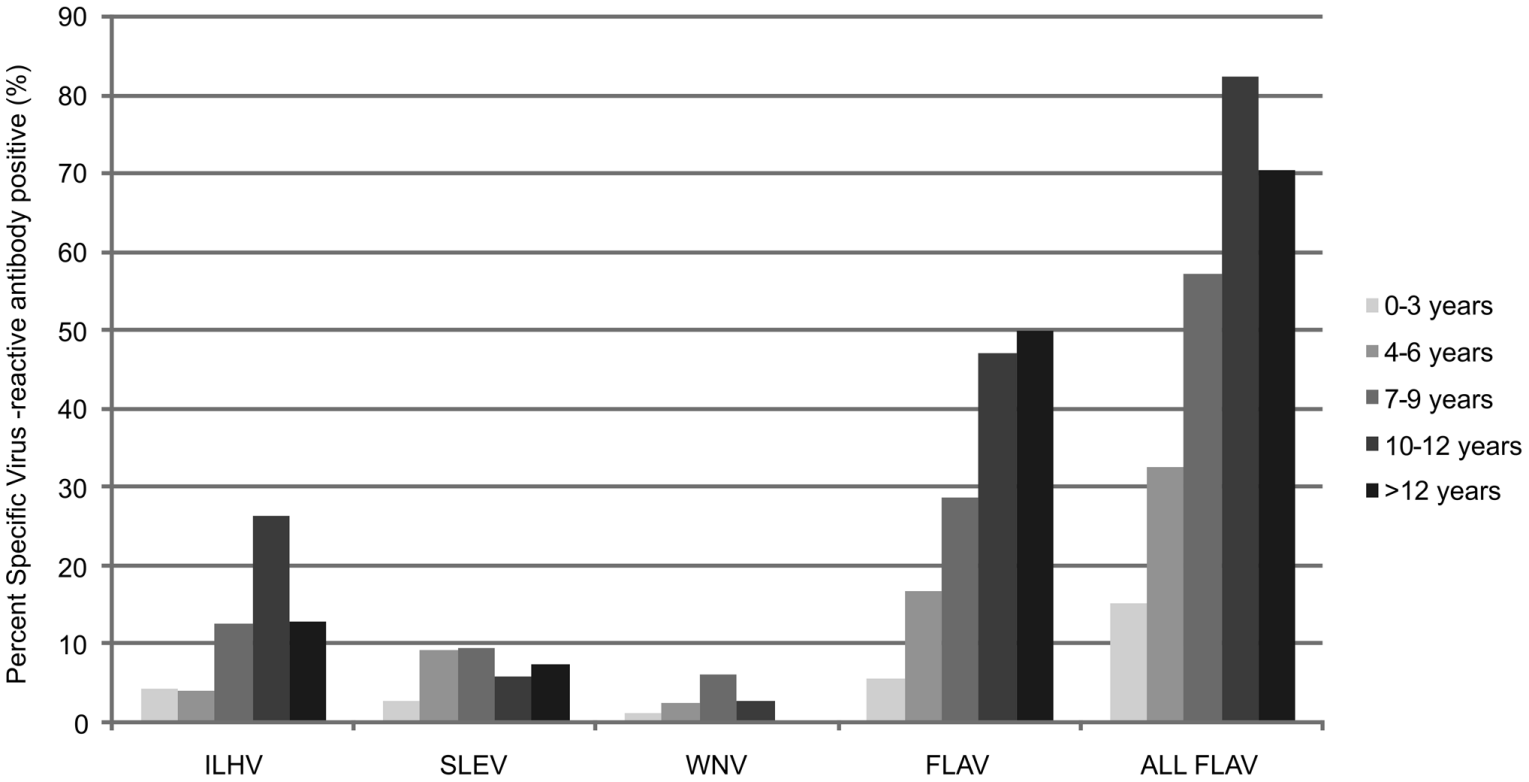
Positive results of PRNT_90_ for flaviviruses in Pantanal equines with known age in years. ILHV: Ilheus virus; SLEV: Saint Louis encephalitis virus; WNV: West Nile virus; FLAV: undifferentiated flavivirus; All FLAV: all flavivirus infections combined; Trends of increasing seroprevalence with age were detected for ILHV and FLAV, but not SLEV and WNV (see text).

From 760 equines tested, 441 (58%) had date of birth available. Stratifying the serology results by age demonstrated that risk of exposure to flaviviruses increased with age for flaviviruses in general and for ILHV (1-tailed p<0.001 for both), but not for SLEV (p = 0.17) or WNV (p = 0.46) (Figure 3). SLEV and WNV appeared to increase in prevalence with age for equines <9 years of age, but not for older animals.

Equines seropositive for CPCV and ROCV were sampled in October 2009. The two CPCV-seropositive horses were five and seven years of age and were from ranch PQ, which comprises an area of approximately 140 square-km. The only horse that was seropositive for ROCV had no record of birth date and was from ranch PM that encompasses a 360-square-km area.

At the end of 2009, an outbreak of neurologic disease among horses was reported from ranch PV located in the Nabileque Sub-region. From 36 equines sampled, 23 (63.8%) were seropositive for flavivirus infection by blocking ELISA. When this subset was tested by PRNT_90_, 11 (30.5%) confirmed seropositive for SLEV, nine (25.0%) for undifferentiated flavivirus(es), two (5.5%) for WNV, and one (2.8%) for ILHV (Table 4). Of the nine undifferentiated flavivirus infections, five had SLEV titers >20 and five had higher SLEV titers than other flaviviruses in the panel, indicating that a majority of these could have had a history of SLEV infection. In addition to the relatively low seroprevalence for WNV of just 5.5% in this sample of 36 equines, four equines from this group that recovered from the recent neurologic syndrome were serologically negative for WNV (Table 4). In spite of recent vaccination against equine encephalitis viruses, most equines had low titers for equine encephalitis viruses. These results will be described separately.

**Table 4:** Equines of a ranch, where an epizootic of undiagnosed neurological syndrome was reported in 2009 and 2010, located in the Nabileque Sub-region of the Pantanal, Brazil.

ID	ELISA	WNV	SLEV	ROCV	ILHV	DENV-1	DENV-4	YFV	IGUV	BSQV	CPCV	DENV-2	DENV-3	RESULT
1039	32.3%	<20	20	<10	<10	<10	<10	<10	<10	<10	<10	<10	<10	FLAV
1046	46.6%	<20	<20	<10	20	<10	<10	<10	<10	<10	<10	<10	<10	FLAV
1052	57.7%	40	80	<10	<10	<10	<10	<10	10	<10	<10	<10	<10	FLAV
1059	30.3%	<20	<20	<10	10	<10	<10	<10	<10	<10	<10	<10	<10	FLAV
1060	77.4%	40	320	80	20	<10	<10	<10	10	10	160	<10	<10	FLAV
1061*	52.2%	<20	40	<10	20	<10	<10	<10	<10	<10	<10	<10	<10	FLAV
1068	58.5%	20	160	<10	80	<10	<10	<10	10	<10	10	<10	<10	FLAV
1078	39.8%	<20	40	10	80	<10	<10	<10	<10	<10	<10	<10	<10	FLAV
1085	38.5%	160	<20	<10	80	<10	<10	<10	10	<10	40	<10	<10	FLAV
1070	41.5%	20	<20	<10	80	<10	<10	<10	10	<10	20	<10	<10	ILHV
1042	56.3%	<20	160	<10	<10	<10	<10	<10	<10	<10	10	<10	<10	SLEV
1054	60.0%	20	160	10	20	<10	<10	<10	<10	<10	40	<10	<10	SLEV
1063	59.9%	20	160	20	20	<10	<10	<10	<10	<10	40	<10	<10	SLEV
1065	57.6%	<20	80	10	10	<10	<10	<10	<10	<10	<10	<10	<10	SLEV
1071	62.0%	<20	40	<10	10	10	<10	<10	<10	<10	10	<10	<10	SLEV
1072*	79.8%	20	1280	40	40	<10	<10	<10	10	<10	40	10	<10	SLEV
1073*	41.7%	<20	320	<10	10	<10	<10	<10	<10	<10	<10	<10	<10	SLEV
1079	37.0%	<20	80	<10	10	<10	<10	<10	<10	<10	<10	<10	<10	SLEV
1081	38.0%	<20	40	<10	10	<10	<10	<10	<10	<10	<10	<10	<10	SLEV
1082	46.8%	<20	40	<10	10	<10	<10	<10	<10	<10	<10	<10	<10	SLEV
1084	51.2%	<20	160	<10	20	<10	<10	<10	<10	<10	<10	<10	<10	SLEV
1080	58.1%	320	80	<10	20	<10	<10	<10	<10	<10	40	<10	<10	WNV
1088	71.6%	40	<20	<10	10	<10	<10	<10	10	<10	<10	10	<10	WNV
1041	9.2%	-	-	-	-	-	-	-	-	-	-	-	-	
1043	17.3%	-	-	-	-	-	-	-	-	-	-	-	-	
1047	13.0%	-	-	-	-	-	-	-	-	-	-	-	-	
1048	16.2%	-	-	-	-	-	-	-	-	-	-	-	-	
1050	15.2%	-	-	-	-	-	-	-	-	-	-	-	-	
1053	8.2%	-	-	-	-	-	-	-	-	-	-	-	-	
1056	16.8%	-	-	-	-	-	-	-	-	-	-	-	-	
1057	23.9%	-	-	-	-	-	-	-	-	-	-	-	-	
1066	11.2%	-	-	-	-	-	-	-	-	-	-	-	-	
1069	3.0%	-	-	-	-	-	-	-	-	-	-	-	-	
1074*	23.3%	-	-	-	-	-	-	-	-	-	-	-	-	
1086	12.4%	-	-	-	-	-	-	-	-	-	-	-	-	
1087	-4.0%	-	-	-	-	-	-	-	-	-	-	-	-	

BSQV, Bussuquara virus; CPCV, Cacipacore virus; ILHV, Ilheus virus; ROCV, Rocio virus; SLEV, Saint Louis encephalitis virus; WNV, West Nile virus; IGUV, Iguape virus; YFV, Yellow fever virus; DENV, Dengue virus; ID: Horse identification; -, Not tested.

* Equines that showed disease during the epizootic and survived.

## Discussion

In the absence of direct viral detection, diagnosis of arbovirus infections is performed by indirect serological tests. However, cross-reactive antibody responses to closely related flaviviruses can complicate interpretation of serologic test results [36]. In particular, secondary flavivirus infections generate high levels of cross-reactive antibodies, which further complicate the interpretation of flavivirus serology. The cross-reacting antibodies of primary infections typically react with other members of the Japanese encephalitis virus serogroup comprised by WNV, CPCV, SLEV and others principally in Africa and Asia [37], [38], but antibodies generated by secondary flavivirus infections can also bind and neutralize viruses of other serogroups as well. Moreover, some individuals that are sequentially infected by a heterologous flavivirus species can boost antibody levels against the original virus, resulting in a phenomenon known as ‘original antigenic sin’ which can lead to incorrect diagnoses [39]. Finally, the potential circulation of unknown flaviviruses in the region could generate cross-reacting neutralizing antibodies, and lead to misinterpretation. Therefore, we used a conservative threshold for detection of neutralizing antibodies (90%) and we considered homotypic serologic responses to be the most reliable, as these samples reacted with just one of the 12 viruses employed in the tests, with no indication of cross-reaction.

In the present study, serological evidence of ILHV, SLEV, WNV, CPCV and ROCV circulation was detected in the Nhecolândia and Nabileque sub-regions of Pantanal. Nhecolândia is the second largest sub-region of Pantanal comprising roughly 27,000 square-km or 19% of the total Pantanal area and it is the world's largest and most diverse area of subtropical lakes [40], [41]. Nabileque is the fifth largest sub-region comprising roughly 14,000 square-km or 10% of the total Pantanal area. This sub-region is formed by fluvial plains of the Paraguay River and remains flooded for nine months or more during the year [42].

The seroprevalence of ILHV and all flaviviruses combined increased with age in equines (Figure 3). This temporal pattern implies low-level enzootic transmission, where risk of infection increases with time. A drop in seroprevalence in the oldest age category that was observed consistently for all the flaviviruses evaluated may be explained by either waning of detectable antibodies over time, and/or weakening of the humoral immune response in older animals. In the case of SLEV and WNV, seroprevalence appeared to increase with age in the youngest three age categories, through age 9 years old, but the sample sizes were insufficient to detect a statistically significant trend. These increases in the youngest age groups may be explained by one or more introductions with establishment or not of these viruses after 2000 (i.e. within 9 years of the sampling time point in 2009–2010). This finding corroborates the negative results found in a serosurvey conducted with local equines in the 1990s, when no neutralizing antibodies for SLEV were detected [24]. These data also corroborate the results of two other equine serosurveys conducted in the Nhecolândia Sub-region in February 2007 and February 2009, when neutralizing antibodies to SLEV were detected in three and four year old equines, respectively, which suggests that SLEV circulated between 2004–2007 [25] and 2005–2009 [21], coinciding with two human outbreaks caused by SLEV in Argentina in 2005 [43] and southeast Brazil in 2006 [4].

In the present study, the detection of SLEV- and ILHV-seropositive geldings both born in September 26, 2009 and sampled on September 30, 2010 with no history of travel outside of Pantanal indicates that the transmission of SLEV and ILHV had occurred as recently as late 2009 or 2010. The enzootic status of ILHV is corroborated by the concurrent isolation of ILHV from *Aedes scapularis* specimens captured in the Nhecolândia Sub-region in 2010 [44].

Regarding WNV, the youngest seropositive equine was a 2 year-old untamed mare with no history of travel. Because it was sampled on October 23, 2009, the infection occurred between 2007 and 2009.

The confirmed WNV-seropositive equines were detected in ten ranches comprising an area of approximately 2,500 square-km in the Nhecolândia and Nabileque sub-regions of the Pantanal, located in MS. Monotypic neutralizing reactions for WNV were detected in six ranches encompassing roughly 1,300 square-km, which comprise approximately 20% of the Nhecolândia Sub-region area. WNV-seropositive equines were detected in Nhecolândia and Nabileque ranches located roughly 90 and 100 km distant from the city of Corumbá, MS respectively (Figure 2). Considering the detection of arbovirus activity in the Nabileque Sub-region which borders Bolivia and Paraguay, these two countries should consider our findings for their arbovirus surveillance programs.

The two CPCV-seropositive equines were sampled October 20, 2009 at five years of age and no history of travel outside of Pantanal, which indicates that infection had occurred between 2004 and 2009.

We report here for the first time the detection of ROCV-neutralizing antibodies in the Pantanal region. ROCV, which is an indigenous Brazilian flavivirus, was the causative agent of an extensive encephalitis epidemic in southeastern Brazil in the 1970s [3]. The detection of neutralizing antibodies for ROCV was unexpected, as current transmission events for ROCV are rare. Considering that the only ROCV-seropositive equine had no birth date available, the timing of ROCV transmission could not be clarified. No ROCV-seropositive equines were detected in two previous serosurveys for flaviviruses conducted in the same region [21], [24], which could indicate low circulation or recent introduction of ROCV in the Pantanal.

No seropositivity for BSQV, IGUV, YFV and DENVs was confirmed among the equines tested in the present study. However, except for DENV-3, neutralizing antibodies were found to all the flaviviruses tested. More investigation is needed to understand the potential circulation of these viruses in the Pantanal region, as they may be restricted to ecological cycles for which equines, sheep and caiman fail to serve as indicators. A serosurvey of non-human primates and/or local human residents would be more instructive for these flaviviruses. Testing only equines may provide a biased view of the relative amounts of flavivirus transmission because equines may not mount immune responses to all viruses equally, or they may not attract all vectors equally. For example, they failed to generate primary immune responses to DENV-2 and SLEV [45] and would not attract large numbers of the yellow fever vector mosquitoes, which are primate specialists [46]. However, they are frequently exposed to mosquitoes and often utilized for arbovirus serosurveys elsewhere.

For future flavivirus serosurveys to be held in the Pantanal, not only ILHV, SLEV, WNV, CPCV and ROCV should be included as differential diagnosis, but also those flaviviruses for which neutralizing antibodies titers ≥20 were detected, including BSQV, IGUV and YFV, if a PRNT with a four-fold greater titer criterion is used.

Regarding the 36 equines collected from ranch PV in the Nabileque Sub-region of Pantanal after a neurologic disorder outbreak occurred in 2009 and 2010, no serological evidence was found linking this epizootic to WNV, which is currently the only flavivirus known to be involved in outbreaks of equine neurological disorders in the New World. Three of four survivors of the recent neurologic syndrome had elevated titers for SLEV, and one was the highest SLEV titer detected in the herd, suggesting that the etiologic agent was a flavivirus that triggered a boost of pre-existing SLEV antibody or that SLEV infection may have been associated with the neurologic syndrome as a contributing risk factor perhaps through antibody enhancement. Equines experimentally infected with SLEV have not developed overt disease [47]. However, the possibility that a novel equine-virulent strain of SLEV caused the neurologic disease in horses in Brazil also merits exploration. SLEV genotype VB was recently isolated from the brain of a single horse that died of encephalitis in the state of Minas Gerais, southeast Brazil, in March 2009 [48]. Of the 36 equines tested from ranch PV, 11 (30.6%) were confirmed seropositive for SLEV, the highest seropositivity compared to 13 other ranches where SLEV-seropositive equines were found (and where maximum seroprevalence was 14%) and significantly higher than SLEV seropositivity detected in all Nhecolândia equines combined (48/724, 6.6%) (p<0.001, chi-square test). The true SLEV infection rate is probably underestimated, as several probable SLEV infections could not be differentiated due to masking by cross-reactive antibodies. Ecological differences between Nabileque and Nhecolândia sub-regions, the former formed by fluvial plains of the Paraguay River and the latter mainly comprised of savannah with subtropical lakes, may have contributed to the difference of seroprevalence encountered in these two sub-regions. Also, the Nabileque ranch is the largest ranch sampled encompassing a 900-square-km area, including portions of the plateau bordering the Pantanal. The high seropositivity for SLEV in Nabileque ranch may reflect higher circulation of SLEV in the plateau surrounding Pantanal than in the floodplains of Pantanal.

Amphibians and reptiles may play a larger role in the transmission cycle of arboviruses than previously assumed. Because of their high energy conversion efficiency, ectotherms typically represent a large portion of the vertebrate biomass in many ecosystems, and may therefore greatly influence transmission dynamics, either as virus amplifiers or dilution hosts [49]. Juvenile *Alligator mississippiensis* has been shown to develop sufficient WNV viremia to infect blood-feeding mosquitoes [50], [51]. A recent study reported the first evidence of WNV exposure in wild Morelet's crocodiles (*Crocodylus moreletii*) in Mexico [52].

Because of widespread, high prevalence of the yacare caiman (*Caiman crocodilus yacare*) in the Brazilian Pantanal, about 3.9 million non-hatchling caimans in 1993 [53], and its potential to naturally amplify WNV in the region, in February of 2009 a serosurvey for flaviviruses was conducted with 30 free-ranging caimans and all animals tested negative for flavivirus antibodies. Despite the negative results, in the same study equines temporally and spatially associated with the seronegative caimans showed neutralizing antibodies for ILHV, SLEV, WNV and CPCV [21]. Taking into account these data, in the present study a wider serosurvey for flaviviruses was conducted with 61 free-ranging caimans from the Nhecolândia Sub-region, but all caimans tested negative for flavivirus antibodies by blocking ELISA and also by PRNT_90_ for WNV. The absence of serological evidence of flavivirus circulation in caimans was unexpected. Possible explanations for these findings are: (i) low circulation of flavivirus in caimans, (ii) caimans may not mount immune responses to flaviviruses and, finally, (iii) caimans may not attract local mosquito vectors of flaviviruses.

In a study conducted also in the Pantanal, but in a different sub-region, 80% of mosquitoes caught blood-feeding on caimans were *Culex (Melanoconion) theobaldi* and *Mansonia* spp. [54], species that are not usually reported as flavivirus vectors. However, more recently, ILHV was isolated from *Aedes scapularis*, which was the most prevalent species caught landing or blood-feeding on caimans from the Nhecolândia Sub-region [44].

The absence of serological evidence of flavivirus circulation in sheep by blocking ELISA and the detection of only two samples with neutralizing antibodies for WNV in very low titers was also an unexpected result, considering that the tested sheep were temporally and spatially associated with equines that were seropositive for various flaviviruses. However, reports of serological evidence of WNV infection in sheep are scarce and have been showing low prevalence [55], [56]. Possible explanations for these findings are the low prevalence of Brazilian flaviviruses in sheep, or that sheep fail to develop antibodies for Brazilian flaviviruses following exposure by mosquito bite, or finally, that sheep may not attract local mosquito flavivirus vectors in the Pantanal region. An entomological study conducted in southeast Brazil in the 1980s showed that sheep was not an attractive host for mosquitoes compared with man, horse and cattle [57].

### Conclusion

In the present study, serological evidence of ILHV, SLEV, WNV, CPCV and ROCV circulation was detected in equines of the Pantanal. Severe disease has been linked to these viruses in humans [7], [13], [43], [58], [59] and for WNV also in equines [60]. WNV and SLEV circulation in the Pantanal appears to be a recent phenomenon. In the last three decades, the Pantanal has been impacted by the conversion of natural vegetation into agricultural fields and cattle pasture, with alteration and loss of natural habitats and biodiversity. Moreover, major negative impacts occur in uplands, with drastic deforestation of savanna vegetation, where main rivers feeding the Pantanal have their springs [22], [61]. These environmental changes in the Pantanal environs can directly impact the fluctuations of vector and vertebrate host populations, which could affect arbovirus transmission dynamics in the region. Loss of wetlands and other natural habitats typically leads to reduced biodiversity, and consequently increased risk of outbreaks of vector-borne diseases [62]. Preserving large wetland areas sustains biodiversity and may represent a valuable ecosystem-based approach for controlling WNV outbreaks [63]. Indeed, an outbreak of neurologic disease among equines reported herein occurred in a ranch that encompasses drier plateau ecosystem on the periphery of the Pantanal wetlands. Serology data suggest that an unknown flavivirus closely related to SLEV or a newly recognized equine-virulent strain of SLEV may be associated with this neurologic syndrome.

Considering the highly conservative criteria used in the present study, the detection of seropositive equines for ILHV, SLEV, WNV, CPCV and ROCV is strong evidence for local circulation of these flaviviruses in the Pantanal of MS, Brazil. These flaviviruses should be included as differential diagnosis in future flavivirus serosurveys to be held in the region. For PRNT studies, not only these flaviviruses, but also YFV, BSQV and IGUV should be included.

It appears that WNV circulated recently in the Pantanal. The detection of WNV-seropositive equines in at least ten ranches is strong evidence of recent widespread circulation, but not necessarily establishment of WNV in the region. Circulation of WNV in this area has not yet been confirmed by virus isolation. Because detection of antibodies is indirect evidence of flavivirus circulation and because novel flaviviruses may exist in the region, we encourage efforts to isolate viruses to confirm the circulation of these flaviviruses. In future investigations of equine epizootics, efforts should be concentrated on the collection of samples suitable for viral isolation.
